# Autophagy in peritoneal fibrosis

**DOI:** 10.3389/fphys.2023.1187207

**Published:** 2023-05-15

**Authors:** Hong-yong Su, Jia-jie Yang, Rong Zou, Ning An, Xiao-cui Chen, Chen Yang, Hai-juan Yang, Cui-wei Yao, Hua-feng Liu

**Affiliations:** Guangdong Provincial Key Laboratory of Autophagy and Major Chronic Non-communicable Diseases, Key Laboratory of Prevention and Management of Chronic Kidney Disease of Zhanjiang City, Institute of Nephrology, Affiliated Hospital of Guangdong Medical University, Zhanjiang, Guangdong, China

**Keywords:** autophagy, human peritoneal mesothelial cells, peritoneal dialysis, peritoneal fibrosis, peritoneal dialysis-related peritonitis

## Abstract

Peritoneal dialysis (PD) is a widely accepted renal replacement therapy for patients with end-stage renal disease (ESRD). Morphological and functional changes occur in the peritoneal membranes (PMs) of patients undergoing long-term PD. Peritoneal fibrosis (PF) is a common PD-related complication that ultimately leads to PM injury and peritoneal ultrafiltration failure. Autophagy is a cellular process of “self-eating” wherein damaged organelles, protein aggregates, and pathogenic microbes are degraded to maintain intracellular environment homeostasis and cell survival. Growing evidence shows that autophagy is involved in fibrosis progression, including renal fibrosis and hepatic fibrosis, in various organs. Multiple risk factors, including high-glucose peritoneal dialysis solution (HGPDS), stimulate the activation of autophagy, which participates in PF progression, in human peritoneal mesothelial cells (HPMCs). Nevertheless, the underlying roles and mechanisms of autophagy in PF progression remain unclear. In this review, we discuss the key roles and potential mechanisms of autophagy in PF to offer novel perspectives on future therapy strategies for PF and their limitations.

## 1 Introduction

To date, more than 2 million people suffer from end-stage renal disease (ESRD), and this prevalence continues to increase year by year, leading to a significant economic burden (Howell et al., 2019; Masola et al., 2022). More than 272,000 patients, approximately 11% of the dialysis population, undergo peritoneal dialysis (PD) worldwide (Balzer, 2020). PD is a common renal replacement therapy for patients with ESRD. Owing to advantages such as simple operation, protection of residual renal function, and lower risk of cross-infection, PD offers a higher quality of life and more cost savings than does hemodialysis for patients with ESRD (Mehrotra et al., 2011; Yeates et al., 2012). During the COVID-19 pandemic, the importance of PD was revealed, especially in children or patients from low-income areas. The peritoneum is the first barrier preventing microbial invasion during the peritoneal fluid exchange. Owing to their large surface and dense vascularization, peritoneal membranes (PMs) can facilitate high solute and water transport to the intraperitoneal region and thus are a natural choice for membrane filtration and removal of excess water and uremic and other solutes (Balzer, 2020).

In patients undergoing long-term PD, the peritoneum is repeatedly exposed to a non-physiological environment with high glucose, high permeability, and low pH. Over time, it develops inflammation, fibrosis, and other peritoneal complications, leading to morphological and functional changes in the PMs, resulting in PM injury and ultrafiltration failure (UF) (Davies et al., 1996; Smit et al., 2004; Masola et al., 2022). UF gradually declines within 2–4 years after the initiation of PD (Davies et al., 1996; Smit et al., 2004; Hayat et al., 2021). Initially, the primary causes of PD failure are peritonitis and catheter-related complications (Li et al., 2016a; Hayat et al., 2021). However, the bio-incompatibility of peritoneal dialysis solution (PDS) in the long term, accompanied by impairment of peritoneal integrity and functionality, becomes the main concern (Bajo et al., 2017). In 50%–80% of the patients undergoing long-term PD, the primary signs of peritoneal fibrosis (PF) are detected within 1–2 years (Strippoli et al., 2016). Inhibition of fibrosis and inflammation can prolong the life of PD therapy. Although many mechanisms have been proposed, effective interventions and treatment strategies for PF are lacking (Zhang et al., 2018a). Thus, there is an urgent need to explore the mechanisms underlying PF progression and develop effective prevention strategies.

Autophagy, a process that degrades single proteins and large organelles, is an integral part of all eukaryotic cell types. It is considered a true health modifier (Morishita and Mizushima, 2019; Klionsky et al., 2021) and serves as the primary regulator of cellular and tissue adaptation to various endogenous and exogenous pressures (Morishita and Mizushima, 2019). Autophagy pathways are physiologically correlated, even under basal and non-stressful conditions. In multiple experimental models, pharmacological and genetic interventions that impair autophagy were found to promote or exacerbate diseases (Klionsky et al., 2021). Autophagy plays a critical role in the progression of fibrosis in various organs, such as the kidneys (Dai et al., 2022), liver (Sun et al., 2021), and lungs (Araya et al., 2013). However, the causal connection between autophagy and PF and the pathophysiological mechanisms remains unclear.

Currently, many researches have proved that autophagy is involved in the process of PD and in PD-related complications, and novel interventions targeting autophagy may have clinical transformability. In this review, we highlight the current knowledge about the key roles and potential mechanisms of autophagy in PF. Furthermore, we discuss novel underlying mechanisms of autophagy modulators, which may contribute to developing new clinical therapies for PF.

## 2 Effect of autophagy in peritoneal fibrosis

PF is one of the most common complications in patients undergoing long-term PD and one of the leading causes of PD failure (Krediet and Struijk, 2013; Krediet, 2018). Epithelial-mesenchymal transition (EMT) of peritoneal mesothelial cells (PMCs), also called mesothelial-mesenchymal transition (MMT), is wildly accepted as the main cause of PF (Strippoli et al., 2016; Zhou et al., 2016; Zhang et al., 2018a; Hayat et al., 2021). Furthermore, in submesothelial areas, transformed mesothelial cells can generate extracellular matrix (ECM) and cause fibrosis, demonstrating their invasive capacity (Yáñez-Mó et al., 2003). Moreover, during PF and MMT, the key fibrogenic molecular machinery, mainly transforming growth factor-β (TGF-β)/Smad-dependent signaling (Margetts et al., 2005; Patel et al., 2010; Loureiro et al., 2011; Yoshizawa et al., 2015) and TGF-β/Smad-independent signaling (Matsuo et al., 2006; Patel et al., 2010; Liu et al., 2012; Fan et al., 2013; Kitterer et al., 2015; Wynn and Vannella, 2016; Padwal et al., 2018), triggers the transcription factors that act on the promoter regions of the cell matrix genes to activate their transcription via specific downstream intracellular signaling.

PF is commonly observed in patients with mild fibrosis undergoing PD (Di Paolo and Sacchi, 2000). However, encapsulating peritoneal sclerosis (EPS), which has low morbidity and high mortality, is an uncommon form of PF (Strippoli et al., 2016). It is a life-threatening complication that may progress even in patients who have discontinued PD.

### 2.1 Effect of autophagy in peritoneal fibrosis

**FIGURE 1:**
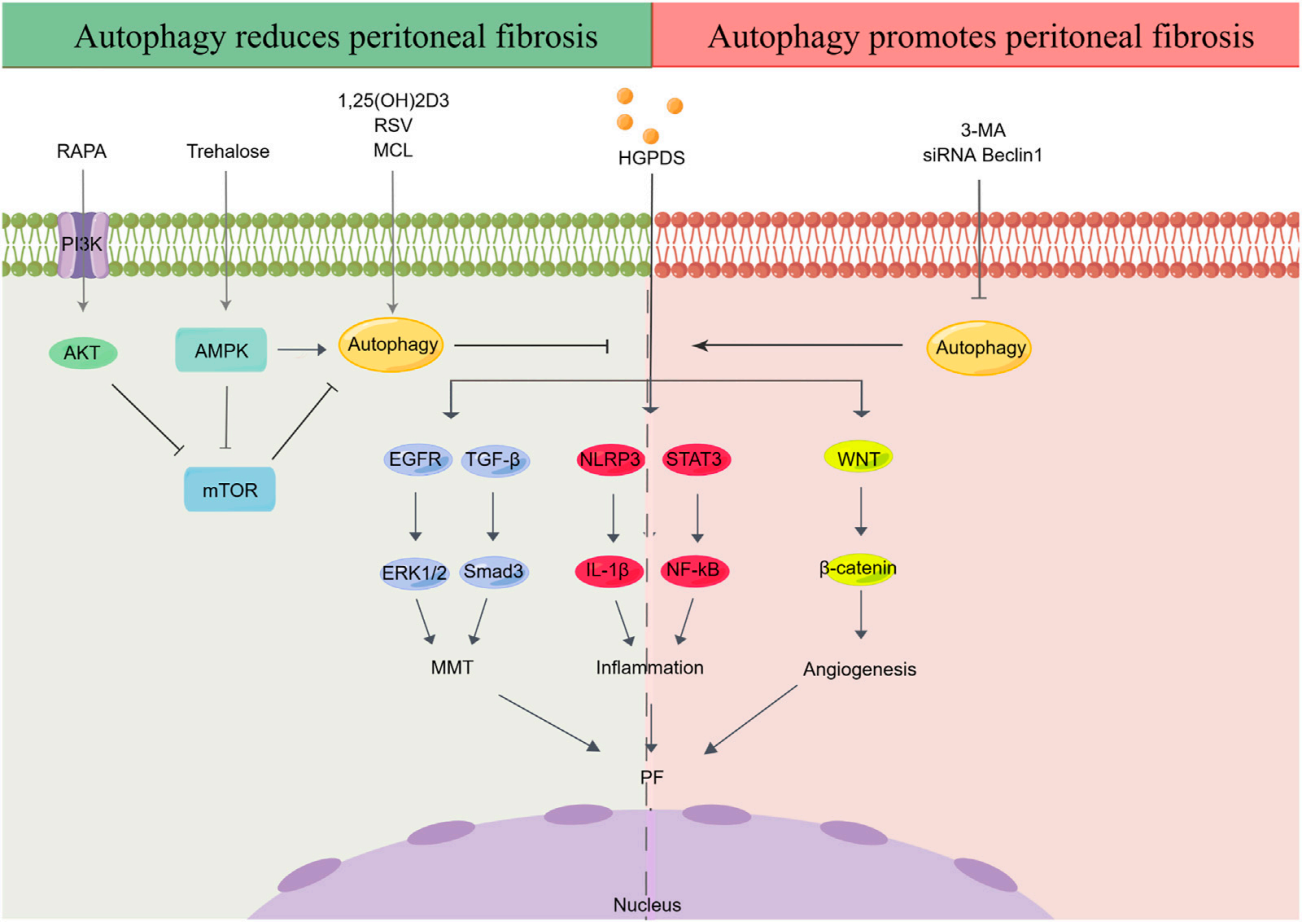
Effect of autophagy in peritoneal fibrosis. HGPDS contributes to MMT, inflammation and angiogenesis in peritoneal mesothelial cells, eventually cause PF. Some studies suggest that activation of autophagy reduces PF, whereas some evidences demonstrate that autophagy promotes PF. RAPA: rapamycin, RSV: resveratrol, MCL: micheliolide, HGPDS: high-glucose peritoneal dialysis solution, 3-MA: 3-methyladenine, PI3K: phosphatidylinositide 3-kinases, AMPK: AMP-activated protein kinase, mTOR: mammalian target of rapamycin, EGFR: epidermal growth factor receptor, ERK1/2: extracellular signal-regulated kinase 1/2, TGF-β: transforming growth factor-β, NLRP3: nod-like receptor 3, IL-1β: interleukin-1β, STAT3: signal transducer and activator of transcription 3, NF-κB: nuclear factor kappa-B, MMT: mesothelial-mesenchymal transition.

As a complex and necessary metabolic process, autophagy exists in most mammalian cells. Autophagy is closely related to fibrosis in multiple organs, such as the kidney (Zhao et al., 2019), heart (Zhao et al., 2018), lungs (Cabrera et al., 2015), and liver (Meng et al., 2018). Additionally, high-glucose (HG) levels can modulate autophagy in multiple disease models (Wei et al., 2014; Chang et al., 2015; Lu et al., 2015). However, the potential effects of autophagy during the development of PF remain ambiguous, as some studies suggest that autophagy reduces PF, whereas others show the opposite trend (Figure 1).

On the one hand, autophagy is believed to contribute to PF progression. First, autophagy inhibition was observed in the peritoneum of an HG-induced peritoneal injury mouse model (Li et al., 2019a). Simultaneously, studies suggest that blocking autophagy with 3-methyladenine (3-MA) and Beclin-1 or ATG5 small interfering RNA (siRNA) not only enhances the continuous activation of the inflammatory factor nod-like receptor 3 (NLRP3)/interleukin-1β (IL-1β) induced by the stimulation of long-term HGPDS but also promotes MMT progression in the PMs of patients undergoing PD (Li et al., 2017). Moreover, as an autophagy inducer, trehalose has been shown to ameliorate PF by promoting the generation of autophagosomes and suppressing MMT in PMCs (Mayer et al., 2016; Sakai et al., 2017; Miyake et al., 2020) through activating AMP-activated protein kinase (AMPK) pathway, phosphorylating unc-52-like kinase-1 (ULK1) at Ser317, and AMPK/mammalian target of rapamycin (mTOR)pathway, dephosphorylating the inhibitory site of ULK1 (Ser757) (DeBosch et al., 2016; Mayer et al., 2016). Under HG conditions, autophagy inhibition has been observed in peritoneal mesothelial cells and mouse models, with reduction in the expression of light chain 3 (LC3)-II, p62, and Beclin-1; contrastingly, 1,25(OH)2D3 alleviates autophagy inhibition in PMCs through the mTOR pathway (Yang et al., 2017). Increasing evidence has shown that regulation of autophagy through the phosphatidylinositol 3-kinase (PI3K)/AKT/mTOR signaling contributes to the occurrence and pathological progression of diabetic nephropathy and PF (Li et al., 2016b; Lu et al., 2019; Yang et al., 2020a; Jia et al., 2022). In this research, treatment with the mTOR inhibitor rapamycin (RAPA) and PI3K inhibitor LY294002 activated autophagy and alleviated PF *in vivo* and *in vitro*, thereby upregulating E-cadherin and zonula occludens-1 (ZO-1) and downregulating alpha-smooth muscle actin (α-SMA) and ferroptosis suppressor protein 1 (Jia et al., 2022). In addition, RAPA, which can induce autophagy by inhibiting MTORC1 expression, relieved peritoneal thickening, angiogenesis, lymphangiogenesis, MMT, and endothelial-to-mesenchymal transition (Endo-MT) and improved UF in a mouse PD model (González-Mateo et al., 2015). Finally, micheliolide (MCL), a natural guaianolide sesquiterpene lactone, which promoted autophagy in db/db mice at a low dose, inhibited TGF-β1-induced ECM accumulation by activating autophagy in PF mouse models and the HPMC cell line (HMrSV5) (Zhong et al., 2018; Li et al., 2019b).

Limited evidence has demonstrated that autophagy promotes PF. Apigenin, a dietary flavonoid synthesized in multiple plants, effectively inhibits PF in a HG-induced mouse model, accompanied by a corresponding alteration of autophagy biomarkers (Zhang et al., 2018a). Moreover, significant activation of autophagy was observed in the PMCs of both two PF rat models induced by PDS and chlorhexidine gluconate (CG) (Shi et al., 2021). However, in above PF rat models and cultured HPMCs, treatment with 3-MA effectively delayed MMT and prevented PF by TGF-β/Smad3 signaling pathway and alleviated peritoneal angiogenesis by downregulation of β-catenin signaling pathway (Shi et al., 2021). Autophagy inhibition significantly reduced MMT, fibrosis, and apoptosis in HPMCs (Wu et al., 2018)(Table 1).

**TABLE 1:** Autophagy in peritoneal fibrosis.

Patients	Animals	Cells	Perineal fibrosis models	Fibrosis	Drugs/compound to modulate autophagy	Autophagy activity	References
NO	-	HMrSV5	HGPDS	↓	RSV,3-MA, ATG5 siRNA, Beclin1 siRNA	↑	Li et al. (2017)
NO	C57BL/6 mice	-	CG	↓	Trehalose	↑	Miyake et al. (2020)
NO	Kunming Mice	HPMCs	HGPDS	↓	1,25(OH)_2_D_3_	↑	Yang et al. (2017)
NO	Rats	Rat PMCs	HGPDS	↓	PI3K inhibitor LY294002 and rapamycin	↑	Jia et al. (2022)
NO	C57BL/6 mice	Patients MCs	HGPDS	↓	Rapamycin	↑	González-Mateo et al. (2015)
NO	C57BL/6 mice	HMrSV5	HGPDS	↓	MCL, rapamycin, ATG7 siRNA	↑	Li et al. (2019b)
NO	BALB/c mice	Mice MCs	HGPDS	↓	Apigenin	↓	Zhang et al. (2018a)
NO	Sprague-Dawley rats	HPMCs	HGPDS and CG	↓	3-MA	↓	Shi et al. (2021)
YES	-	MET-5A	HGPDS	↓	Beclin1 siRNA	↓	Wu et al. (2018)

Similar dual characteristics of autophagy have been demonstrated in renal (Liang et al., 2022) and hepatic fibrosis (Sun et al., 2021; Hou et al., 2022). The effect of autophagy activation in the process of PF is complex and multifactorial, and its molecular impact may vary from specific targets in autophagy regulation. Furthermore, different experimental PF models, cell categories, drugs, doses and timing of autophagy inducers, and the diversified molecular mechanisms could result in varying effects of autophagy on PF. It is generally accepted that autophagy functions differently in diverse diseases and at different stages of the same disease. Over-activation of autophagy promotes fibrosis of multiple organs, whereas inhibition of autophagy aggravates cell damage and promotes the occurrence of PF. Nevertheless, further studies are needed to explore whether the different factors mediating autophagy play different roles at different stages of PF. Autophagy activation is usually considered effective in acute pathological damage to maintain cell homeostasis. In contrast, sustained autophagy induced by some chronic diseases may be harmful, causing apoptosis by damaging important organelles.

Furthermore, mitophagy may be involved in PF. With the production of reactive oxygen species (ROS), mitochondrial DNA is more prone to mutations than nuclear DNA, which makes mitochondria more susceptible to damage. Thus, maintaining an intact population of mitochondria via quality control mechanisms, including mitophagy, is essential for cell survival under pathological stress conditions (Lemasters, 2005; Youle and Narendra, 2011). Many studies have shown that HPMCs stimulated with HGPDS can undergo oxidative stress, mitochondrial DNA damage, and even apoptosis (Hung et al., 2014; Ramil-Gómez et al., 2021). However, insufficient or excessive autophagy of mitochondria results in the accumulation of damaged mitochondria, which eventually leads to the disruption of mitochondrial quality control and bioenergy metabolism and even cell death (Kubli and Gustafsson, 2012; Namba et al., 2014; Kawakami et al., 2015). The mitochondrial dysfunction subsequently participates in the occurrence and development of fibrosis (Li et al., 2020). Under long-term hypertonic and HGPDS, HPMCs exhibit progressive PF, and MMT of HPMCs caused by mitochondrial dysfunction is one of the possible mechanisms (Shin et al., 2017; Ramil-Gómez et al., 2021). Furthermore, metformin and other AMPK inhibitors could delay the phenotypic transition of PMCs and PF via modulating oxidative stress, suggesting that AMPK could be a novel therapeutic target to prevent PF. However, there is no direct evidence that mitophagy can inhibit the progression of PF, which requires further experimental proof (Shin et al., 2017).

### 2.2 Effect of autophagy in encapsulating peritoneal sclerosis

Autophagy may have a potential connection with EPS, a rare but severe complication of patients undergoing PD (Pepereke et al., 2022). EPS, a clinical syndrome characterized by the formation of a fibrous cocoon in the peritoneal cavity, has a high mortality rate of 42% 1 year after diagnosis (Brown et al., 2009). Although there are few treatment methods, and their efficacy is poor. Anti-inflammatory therapy with steroids and antifibrotic therapy with tamoxifen are most commonly used for treating EPS (Lo and Kawanishi, 2009; Garosi et al., 2013; Pepereke et al., 2022).

Recently, mTOR inhibitors (everolimus and sirolimus), widely used as immunosuppressive/antiproliferative agents in renal transplantation and oncology field, have been suggested as a novel treatment for EPS. Two *in vivo* experiments indicated that oral mTOR inhibitors (everolimus and sirolimus) significantly reduced the progression of PF, including peritoneal thickness, vascularity, and fibrosis score, in a rat model induced by CG (Duman et al., 2008; Ceri et al., 2012). Furthermore, mTOR inhibitors may protect the peritoneum from PF by inhibiting MMT, with an upregulation of the E-cadherin and downregulation of α-SMA (Aguilera et al., 2005). In addition, multiple clinical studies have proposed the therapeutic role of mTOR inhibitors in the management of EPS owing to the antifibrotic and anti-angiogenesis effects. Due to the rarity of EPS, most studies come from case reports rather than systematic research. In particular, Ghadimi et al. reviewed the effects of mTOR inhibitors (everolimus and sirolimus) in the therapy of EPS using 13 case reports (Ghadimi et al., 2016). In one-third of the patients with EPS, mTOR inhibitors were found to allay EPS and reduce mortality. Moreover, 5-aminoimidazole-4-carboxyamide ribonucleoside (AICAR), a classical AMPK agonist that reduces inflammation, fibrosis, and cellular ROS injury and promotes autophagy, inhibits postoperative adhesion formation *in vivo* and *in vitro* by inhibiting inflammation and oxidative stress response and accelerating peritoneal mesothelial cell repair (Wu et al., 2022).

The therapeutic effects and mechanism of autophagy against EPS have not been fully confirmed, and more evidence is needed.

## 3 Effect of autophagy in PD-related peritonitis

**FIGURE 2:**
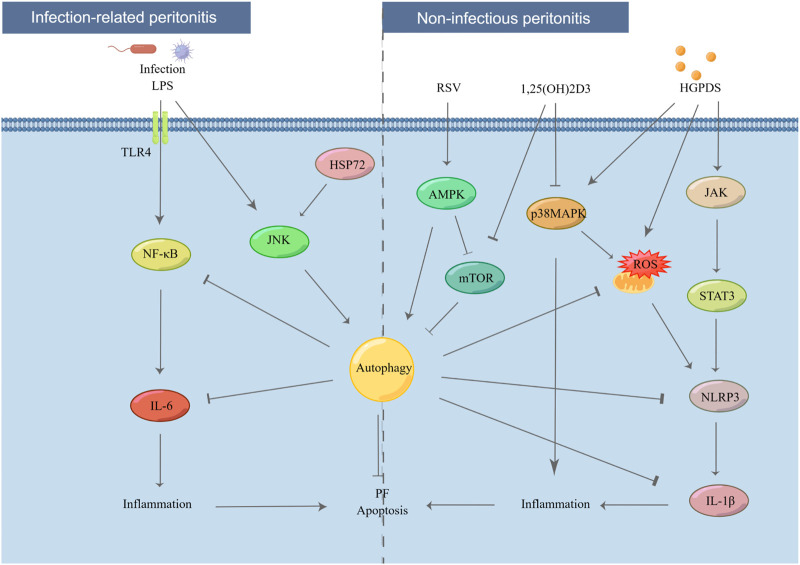
Effect of autophagy in PD-related peritonitis. Infectious factors lead to infection-related peritonitis via NF-κB/IL-6 pathway. In addition, under long-term stimulation of HGPDS, HPMCs suffer from non-infectious peritonitis by NLRP3/IL-1β pathway. However, these processes are blocked by the activation of autophagy. LPS: lipopolysaccharide, TLR4: toll like receptor 4, IL-6: interleukin-6, JNK: c-Jun N-terminal kinase, HSP72: heat shock protein 72, p38 MAPK: p38 mitogen activated protein kinases, JAK: Janus kinase.

PF encompasses two synergistic actions: the fibrosis process itself and inflammation. These two processes are usually bidirectional, with one triggering another (Yáñez-Mó et al., 2003; Loureiro et al., 2011; Yoshizawa et al., 2015; Zhou et al., 2016). PF often progresses after acute and chronic PD-related inflammatory (Kobayashi et al., 2018). Thus, the effects of autophagy on PD-related peritonitis must be elucidated (Figure 2).

Recently, PD-related peritonitis, the common and serious complication of PD, has attracted increasing public attention. More than 40% of patients experience UF owing to PD-related peritonitis after 3 years of PD therapy (Baroni et al., 2012). Mild systemic inflammation is usually observed in patients with chronic kidney disease (Stenvinkel and Alvestrand, 2002). PD-related peritonitis can be primarily classified into infection-related and non-infectious peritonitis.

On the one hand, a single microorganism is the main cause of the infection-related peritonitis, with half of these infections come from gram-positive bacteria (Peterson et al., 1987; Golper et al., 1996; Gokal, 2002; Yung and Chan, 2012). According to the comprehensive recommendations on the prevention and treatment of PD-related peritonitis by the International Society for PD, appropriate and adequate antimicrobial treatment is still the most important and main therapy for infection-related peritonitis (Szeto and Li, 2019). However, despite apparent clinical remission of peritonitis, the inflammatory factors and fibrotic mediators persistently increased in the peritoneal cavity cause continuous stimulation to PMCs (Yung and Chan, 2012). In addition, evidence indicates that pro-inflammatory factors are continuously released for at least 6 weeks even after the clinical resolution of PD-related peritonitis (Lai et al., 2000; Lai et al., 2007). On the other hand, when repeatedly exposed to a non-physiological condition of HGPDS, PMCs raise an inflammatory response in the peritoneal cavity, which causes non-infectious peritonitis (Lai and Leung, 2010). Research has established that non-infectious peritonitis also results in PF, neoangiogenesis, and UF (Lai et al., 2000; Myers, 2004; De Vriese, 2005; Zareie et al., 2005; De Vriese et al., 2006; Leung et al., 2006; Aroeira et al., 2007; Guo et al., 2007; Lai et al., 2007; Leung et al., 2009).

### 3.1 Effect of autophagy in infection-related peritonitis

Several studies have suggested that autophagy has a strong relationship with both infection-related and non-infectious peritonitis, suggesting that autophagy might act as a novel therapeutic target for patients with PD-related peritonitis.

HPMCs are considered as the first barrier to prevent against invading pathogens in patients on PD. Autophagy plays a key role in innate immunity and cell homeostasis (Gutierrez et al., 2004; Nakagawa et al., 2004; Thurston et al., 2009; Sir et al., 2010; Anand et al., 2011). After bacterial invasion, autophagy is activated, degrading bacteria via the autophagy-lysosomal pathway, thus protecting the host against pathogen colonization (Ligeon et al., 2011; Choi et al., 2013). Autophagy activation plays a critical role in the recovery of mesothelium following acute inflammation in a rat model by removing damaged cytoplasmic organelles (Balogh et al., 2015). Lipopolysaccharide (LPS), the active constituent of endotoxins in gram-negative bacteria, is a potential inducer of autophagy in PMCs and macrophages (Xu et al., 2007; Doyle et al., 2011; Meng et al., 2012). In particular, autophagy is induced by LPS in HMrSV5 in a dose- and time-dependent manner, as demonstrated by an increase in the expression of Beclin-1 and LC3-II, number of autophagic vacuoles, and intracellular bactericidal activity (Li et al., 2011). However, these processes in the peritoneal cavity are blocked by autophagy inhibitors, such as 3-MA, Beclin-1 siRNA, or wortmannin, via TLR4 signaling (Wang et al., 2013). A study suggested that heat shock protein 72 (HSP72) protects peritoneal mesothelial cells against LPS-induced mesothelial cell injury by activating c-Jun N-terminal kinase (JNK)-dependent autophagy and partially inhibiting apoptosis (Li et al., 2011). Furthermore, peroxisome proliferator-activated receptor-γ (PPAR-γ) may act as a potential therapeutic target for peritonitis (Zhang et al., 2018b). With the stimulation of LPS, upregulating the expression of nuclear factor kappa-B (NF-κB) activity, *ICAM-1*, and interleukin (IL)-6 was observed in rat PD models (Zhang et al., 2015). Although fungal peritonitis is rare compared to bacterial peritonitis, accounting for only 1%–12% of PD-related peritonitis (Chavada et al., 2011; Tobudic et al., 2013; Nadeau-Fredette and Bargman, 2015), it has higher morbidity and catheter-related mortality (Levallois et al., 2012; Li et al., 2016a; Giacobino et al., 2016; Xu et al., 2022). *Candida* species are the most common pathogens involved in most fungal peritonitis cases, with approximately 70%–90% frequency (Kazancioglu et al., 2010; Nadeau-Fredette and Bargman, 2015). In addition, scientific evidence suggests that caspase recruitment domain-containing protein 9 (Card9), an adapter protein, protects against fungal peritonitis by regulating the mucosa-associated lymphoid tissue lymphoma translocation 1 (Malt1)-mediated activation of autophagy in macrophages (Xu et al., 2022).

### 3.2 Effect of autophagy in non-infectious peritonitis

Autophagy also contributes to non-infectious peritonitis. PMs develop chronic inflammation during long-term PD as a result of bioincompatible PDS with low pH, high concentrations of glucose, and high osmolality (Hayat et al., 2021). IL-6 is a crucial inflammatory factor in patients with PD, and repeated inflammatory activation can lead to fibrotic injury in the peritoneum via the JAK/Signal Transducer And Activator Of Transcription 3 (STAT3) signaling pathway (Fielding et al., 2014; Xiao et al., 2017; Yang et al., 2020b; Yang et al., 2021). Moreover, evidence indicates that the upregulation of IL-17 family cytokines protects the host from infections and chronic inflammation during PD-associated peritoneal injury (Schroder and Tschopp, 2010; Helmke et al., 2021). Long-term continuous exposure to HG PDS leads to mitochondrial ROS production in HPMCs, which subsequently triggers NLRP3 inflammasome activation and IL-1β secretion (Witowski et al., 2001; Yáñez-Mó et al., 2003; Schroder and Tschopp, 2010; Li et al., 2017). However, ROS can induce autophagy, a self-clearance process that reduces oxidative damage via engulfing and degrading the oxidized substances (Wu et al., 2016; Li et al., 2021). Timely initiation of autophagy could block the activation of NLRP3-IL-1β signaling, providing a theoretical basis for a potential therapeutic strategy to suspend inflammation and PF (Li et al., 2017). Resveratrol-induced mitophagy/autophagy by the AMPK pathway may protect against ROS-NLRP3-mediated inflammatory injury in HPMCs (Wu et al., 2016). Research has conclusively demonstrated that HG treatment leads to apoptosis, ROS production, inflammatory activation, and MMT in PMCs via the MAPK/P38 signaling pathway, while 1,25(OH)2D3 blocks the above-mentioned changes (Zhang et al., 2012; Yang et al., 2016). A recent study found that 1,25(OH)2D3 plays a protective role in HG-induced peritoneal injury by increasing autophagy, possibly via the mTOR signaling pathway (Yang et al., 2017). However, opposing views exist. The blockade of autophagy with 3-MA considerably reduced the inflammatory response and macrophage infiltration via the STAT3/NF-κB pathway in both PDS and CG-induced rat models (Shi et al., 2021).

## 4 Conclusion and perspective

In the last decades, many researchers have suggested a strong relationship between the process of PF and autophagy, but the roles and potential mechanisms are not completely clear, probably due to the versatility of autophagy.

Owing to the continuous research on the role of autophagy in the prevention and treatment of diseases, some drugs targeting autophagy have been used for the treatment of cancer, infection-related diseases, and neuropathy. Meanwhile, drugs targeting autophagy, such as mTOR inhibitors (evolutionus and sirolimus), have also been used in the treatment of EPS. However, most of the clinical data on EPS comes from case reports, while the registries and treatments of EPS provide low-quality evidence owing to its rarity, making it difficult to carry out large-scale systematic research on the role of autophagy in EPS. Histone deacetylase (HDAC) inhibitors or lysosomal acidification inhibitors (such as chloroquine and hydroxychloroquine) may also be used for the regulation of autophagy and are often used in immunotherapy in kidney diseases. However, there is less clinical evidence in PF or peritonitis.

There are several potential aspects of autophagy research in PF. First, the specific mechanism of autophagy in PF and peritonitis needs to be clarified. Selective autophagy, such as mitophagy and macrophagy, may also be involved in PF. Additionally, potential autophagy markers are also attractive. For example, owing to the objective advantages such as simple operation, small invasion, and fast recovery, the ‘pull technology’ for PD catalyst removal is more popular among doctors and patients than the traditional open surgery, which makes it hard to obtain tissue samples. With the simpler, more economical, easier to obtain, and repeatable advantages, peritoneal dialysis fluid, rather than peritoneal tissue, can continuously and remarkably detect autophagy activity and peritoneal fibrosis. Additionally, the application of intervention drugs is equally important. Some autophagy activators can be added to peritoneal dialysis fluid for preventing peritoneal complications. Effective measures should be implemented to promote the transformation of experimental data into clinical practice. Autophagy modulators that can be used to develop novel clinical therapeutic strategies for allaying PF need to be developed to reveal the mechanism of autophagy pathways in PF.
